# Differences in cancer survival among white and black cancer patients by presence of diabetes mellitus: Estimations based on SEER‐Medicare‐linked data resource

**DOI:** 10.1002/cam4.1554

**Published:** 2018-05-23

**Authors:** Clara Lam, Kathleen Cronin, Rachel Ballard, Angela Mariotto

**Affiliations:** ^1^ Division of Cancer Control and Population Sciences National Cancer Institute Rockville MD USA; ^2^ Office of Disease Prevention Office of the Director National Institutes of Health Rockville MD USA

**Keywords:** cancer‐specific survival, diabetes, race, SEER

## Abstract

Diabetes prevalence and racial health disparities in the diabetic population are increasing in the US. Population‐based cancer‐specific survival estimates for cancer patients with diabetes have not been assessed. The Surveillance, Epidemiology, and End Results (SEER)‐Medicare linkage provided data on cancer‐specific deaths and diabetes prevalence among 14 separate cohorts representing 1 068 098 cancer patients ages 66 +  years diagnosed between 2000 and 2011 in 17 SEER areas. Cancer‐specific survival estimates were calculated by diabetes status adjusted by age, stage, comorbidities, and cancer treatment, and stratified by cancer site and sex with whites without diabetes as the reference group. Black patients had the highest diabetes prevalence particularly among women. Risks of cancer deaths were increased across most cancer sites for patients with diabetes regardless of race. Among men the largest effect of having diabetes on cancer‐specific deaths were observed for black men diagnosed with Non‐Hodgkin lymphoma (NHL) (HR = 1.53, 95%CI = 1.33‐1.76) and prostate cancer (HR = 1.37, 95%CI = 1.32‐1.42). Diabetes prevalence was higher for black females compared to white females across all 14 cancer sites and higher for most sites when compared to white and black males. Among women the largest effect of having diabetes on cancer‐specific deaths were observed for black women diagnosed with corpus/uterus cancer (HR = 1.66, 95%CI = 1.54‐1.79), Hodgkin lymphoma (HR = 1.62, 95%CI = 1.02‐2.56) and breast ER+ (HR = 1.39, 95%CI = 1.32‐1.47). The co‐occurrence of diabetes and cancer significantly increases the risk of cancer death. Our study suggests that these risks may vary by cancer site, and indicates the need for future research to address racial and sex disparities and enhance understanding how prevalent diabetes may affect cancer deaths.

## 1 INTRODUCTION

The prevalence of diabetes in the United States (US) has been steadily increasing over the past 25 years^1^ and appears to mirror increasing prevalence of obesity in the US. Diabetes prevalence varies by racial and ethnic groups with non‐Hispanic black and Hispanic adults experiencing the highest rates.^2^, ^3^, ^4^ While mortality rates for many specific cancers and for cancers overall have been improving,^5^, ^6^ disparities in mortality between black and white cancer patients have persisted, and in some cases, have increased.^7^ Many potential hypotheses for these differences have been proposed; however, the possible influence of the much higher prevalence of diabetes among black compared to white cancer patients has not been explored in depth.

Previous studies have investigated the association between preexisting diabetes and the incidence of selected specific cancers^8^, ^9^, ^10^, ^11^, ^12^, ^13^, ^14^ and with long‐term, all‐cause mortality.^15^ Additionally, several studies have investigated possible differences in cancer‐specific survival between white and black cancer patients with and without diabetes, but these studies have been limited to examining a single cancer patient population or have been drawn from more selected patient populations that may be less representative of the overall US cancer patient populations.^16^, ^17^, ^18^, ^19^, ^20^ Furthermore, some studies were conducted outside the US or had small sample sizes.^15^ To date, a general overview of population‐based cancer‐specific survival and risk of cancer death estimates for different cohorts of cancer patients with diabetes by race have not been assessed. Therefore, in this study, we used 2 unique national data resources, the Surveillance, Epidemiology, and End Results (SEER)‐linked Medicare claims data to identify black and white cancer patients 66 years and older, representing more than 50% of new cancer cases, with and without prevalent diabetes to determine differences in the risk of cancer death.^21^

## 2 METHODS

### 2.1 Data source

Cancer patients were identified through SEER‐Medicare, a linkage of 2 large population‐based data sources comprised of the National Cancer Institute funded Surveillance, Epidemiology, and End Results (SEER) registries and Medicare claims.^22^ SEER collects information on persons diagnosed with cancer in designated areas and provides specific demographic, clinical, cancer characteristics, and cause of death information. In this study, we used data from SEER‐17 (November 2014 submission: Connecticut, Hawaii, Iowa, New Mexico, Utah, rural/greater Georgia, California, Kentucky, Louisiana, New Jersey, and metropolitan areas including San‐Francisco‐Oakland, Detroit, Seattle‐Puget Sound, Atlanta, San Jose‐Monterey, and Los Angeles) which cover approximately 30% of the total US population.^23^ Medicare provides federally funded health insurance for persons ages 65 years and older and comprises approximately 45 million people in the US. SEER‐Medicare links 94% of SEER cancer cases diagnosed at ages 65 years and older and includes information on all Medicare covered services for beneficiaries with fee‐for‐service coverage.^22^

### 2.2 Study population

We selected cancer patients aged 66 years or older diagnosed with 14 specific primary only cancers between 2000 and 2011, who had continuous Medicare Part A and Part B enrollment and were not enrolled in an HMO during the year prior to diagnosis to ensure complete Medicare coverage to assess diabetes status. Cancer sites included bladder, cervix, colon/rectum, corpus/uterus, estrogen receptor (ER) positive female breast, ER negative female breast, Hodgkin lymphoma, liver, myeloma, non‐Hodgkin lymphoma (NHL), ovary, pancreas, prostate, and stomach. These sites were chosen because they represent the most common cancers diagnosed among men and women in the US and because previous research have shown high diabetes prevalence and racial disparities among these cancer populations.^6^ We did not include lung cancer in our study because of our inability to assess smoking in SEER‐Medicare data which would have been a major confounder in our analyses that could not be directly measured. Additionally, we wanted to focus on cancer sites that were not well represented in previous studies (due to small sample sizes) or could potentially be related to diabetes or the pathway to the development of diabetes. In addition to the 14 selected cancer cohorts, we also obtained claims information on 100 000 controls, individuals without cancer from a 5% random sample of cancer‐free Medicare recipients in the SEER catchment areas.^22^ The data on individuals without cancer provided background diabetes prevalence information in the general population for comparison to the different cancer cohorts. To estimate the prevalence of diabetes for controls in each calendar year, we frequency‐matched controls to cancer cases by sex and age.^24^ Controls were only sampled once in a calendar year but could be sampled repeatedly across multiple years.

### 2.3 Diabetes ascertainment

Diabetes was identified from Medicare Part A hospitalization claims and Part B physician/supplier and outpatient facility claims per the International Classification of Diseases, 9th edition (ICD‐9) codes. Analyses included only individuals aged 66 years and older and conditions in the physician claims were required to appear more than once in a period greater than 30 days within a 1‐year period from the first claim to be considered a diabetes claim.^25^, ^26^, ^27^ Diabetes (ICD‐9: 250.0x‐2503x, 250.7x) and diabetes with sequelae (ICD‐9: 250.4x‐250.6x, 250.8x‐250.9x) were grouped together.^25^, ^26^ For each cancer patient, diabetes status was identified in the year before first cancer diagnosis, excluding month of diagnosis, to minimize misclassification of complications potentially related to cancer diagnosis or treatment. Diabetes status for individuals without cancer was identified in the year before the birthday of the age at diagnosis of the matched case.

### 2.4 Other variables

Adjuvant cancer treatment included receipt of chemotherapy, radiotherapy, hormonal therapy, or immunotherapy as reported in SEER. Patients who received any of these therapies were classified as having received cancer treatment or as not having had treatment/unknown if had treatment if there were no reports of treatment in the SEER data. Selected comorbidities from the Charlson comorbidity index included chronic obstructive pulmonary disease, mild/moderate/severe liver disease, AIDS, peptic ulcer disease, and rheumatologic disease.^25^, ^26^ Other comorbidities, namely congestive heart failure, moderate/severe renal disease, dementia, history of myocardial infarction, acute myocardial infarction, peripheral vascular disease, hemiplegia/paraplegia, and cerebrovascular disease, were not included because they have been shown to be related to obesity, may be complications of diabetes, or increase risk of death from diabetes and would have resulted in over adjustment of the risk estimates.^28^, ^29^, ^30^, ^31^, ^32^, ^33^, ^34^, ^35^, ^36^, ^37^, ^38^, ^39^, ^40^, ^41^ Because of possible misclassification of causes of death from death certificate we used the SEER cause‐specific death classification variable to classify deaths into cancer and noncancer categories.^42^ This variable uses the sequence of the cancer and causes of death that are likely to be related to the particular cancer or as a consequence of a cancer diagnosis to better classify as a death attributable to the cancer.^43^ Noncancer deaths or lost to follow‐up were considered to be censoring events.

### 2.5 Statistical analysis

We calculated diabetes prevalence by different demographic characteristics among the cancer cases and controls. Age‐adjusted 5‐year cause‐specific survival and 95% confidence intervals (CI) were calculated using SEER*Stat and the SEER cause‐specific death classification variable.

Analyses were restricted to patients diagnosed with cancer between 2000 and 2011 to provide the survival experience of the most recently diagnosed cancer patients.^44^ Patients diagnosed at autopsy, on death certificates, or who had zero survival months were excluded. Cox proportional hazards regression was used to calculate hazard ratios (HR) and 95% CI to assess the effect of diabetes and race, on cancer‐specific deaths.^4^, ^45^, ^46^ We allowed for the possibility that diabetes effects on cancer survival might be varied by race so we calculated separate hazard ratios by including a four‐level variable that combined race and diabetes status, with whites without diabetes serving as the reference group. Whites without diabetes were chosen to be the referent group because they are known to have better survival compared to other groups. With an exception of Hodgkin lymphoma in males and stomach cancer in females, we found statistically significant interactions (*P* < .05) between race and diabetes for all other cancer sites. These analyses were stratified by cancer site and sex. Additionally, analyses were adjusted by age group (66‐69 years, 70‐74 years, 75‐79 years, 80‐84 years, 85+ years), selected comorbidities, receipt of cancer treatment, and stage at cancer diagnosis (localized, regional, distant, or unstaged/unknown). All analyses were conducted using SAS 9.3 (Cary, NC).

## 3 RESULTS

We identified 1 068 098 cancers diagnosed between 2000 and 2011, with female breast, prostate, and colorectal cancer representing the most common cancers (Table 1). The majority of cancer patients were white (91%), less than 80 years old, male, and diagnosed with earlier stage cancers. The percentage of cancer patients with diabetes did not vary substantially by age, sex, and stage at diagnoses. Patients with liver and pancreas cancers had the highest prevalence of diabetes (42.1% and 35.0%, respectively) compared to other cancer cohorts. Black patients had a higher prevalence of diabetes irrespective of cancer status. Although the matched noncancer controls had a slightly lower prevalence of diabetes than the cancer cases (20% vs 22%); the prevalence of diabetes was higher in noncancer controls among blacks than whites and did not differ greatly by age or sex.

**Table 1:** Descriptive characteristics and number and percent with diabetes for 1 068 098 between SEER‐Medicare cancer patients ages 66+ y diagnosed 2000 and 2011 for 14 cancers and matched individuals without cancer in the 5% random sample

Characteristic	All cancers			Individuals without cancer		
	Total	With diabetes		Total	With diabetes	
	N	N	%	N	N	%
Total	1 068 098	237 049	22.2	100 000	19 722	19.7
Age, y						
66‐69	210 141	44 779	21.3	19 673	3,689	18.8
70‐74	267 815	61 610	23.0	25 076	5040	20.1
75‐79	247 548	58 104	23.5	23 176	4899	21.1
80‐84	188 056	42 639	22.7	17 605	3651	20.7
85+	154 538	29 917	19.4	14 470	2443	16.9
Race/ethnicity						
White	973 300	207 537	21.3	91 121	17 094	18.8
Black	94 798	29 512	31.1	8879	2628	29.6
Sex						
Male	564 980	127 070	22.5	52 897	10 984	20.8
Female	503 118	109 979	21.9	47 103	8738	18.6
Stage at diagnosis						
In situ	29 588	7171	24.2	~	~	~
Localized	461 077	98 191	21.3	~	~	~
Regional	194 016	43 991	22.7	~	~	~
Distant	228 192	51 462	22.6	~	~	~
Unknown/unstaged	155 225	36 234	23.3	~	~	~
Cancer sites						
Bladder	60 381	14 199	23.5	~	~	~
Cervix	3,373	743	22.0	~	~	~
Colon/Rectum	125 909	29 531	23.5	~	~	~
Corpus/Uterus	25 247	6488	25.7	~	~	~
Female breast						
ER positive	89 278	17 753	19.9	~	~	~
ER negative	16 817	3510	20.9	~	~	~
Hodgkin lymphoma	1989	497	25.0	~	~	~
Liver	10 157	4276	42.1	~	~	~
Myeloma	15 890	3647	23.0	~	~	~
Non‐Hodgkin lymphoma	44 566	9781	21.9	~	~	~
Ovary	15 419	2948	19.1	~	~	~
Pancreas	34 058	11 928	35.0	~	~	~
Prostate	195 167	36 720	18.8	~	~	~
Stomach	18 056	4945	27.4	~	~	~
Other	411 791	90 083	21.9	~	~	~

ER, estrogen receptor.

A random sample of 100 000 individuals (controls) was chosen by frequency matching to all the sites combined cancer cohort by calendar year, age, and sex.

Controls can be sampled only once in a calendar year but can be sampled repeatedly across multiple years.

Other cancers include all reportable cancers made to SEER not individually specified as a separate cohort in this study.

When stratified by cancer site, black males had a higher prevalence of diabetes than white males, except for liver cancer for which diabetes was present in 44.5% of white males compared to 33.9% of black males (Table 2). Black females had higher prevalence of diabetes across all cancer sites compared to white females. White males generally had better 5‐year age‐adjusted cause‐specific survival compared to black males. Survival rates were similar between white and black male cancer patients for pancreatic and prostate cancer and better for black compared to white male cancer patients for Hodgkin lymphoma, and myeloma. Similar to patterns observed in male cancer patients, white female cancer patients had lower diabetes prevalence, regardless of cancer site, and tended to have better 5‐year age‐adjusted cause‐specific survival. The 5‐year survival estimates were similar between white and black female cancer patients for liver, ovary, and stomach cancers and better for black compared to white female cancer patients for Hodgkin lymphoma and myeloma. In general, the magnitude of difference in the prevalence of diabetes between black and white female cancer cases is much larger (approximately 15% for most cancer sites) than the difference between black and white male cancer cases (<10% for both cancer sites).

**Table 2:** Prevalence of diabetes and 5‐y age‐adjusted cause‐specific survival by cancer site, sex, and race among SEER‐Medicare cancer patients 66+ y diagnosed in 2000‐2011

Cancer site	Race	Males			Females		
		% with diabetes	5‐y age‐adjusted cause‐specific survival (%)	95% CI	% with diabetes	5‐y age‐adjusted cause‐specific survival (%)	95% CI
Bladder	White	24.4	78.6	77.6‐79.5	19.0	68.2	66.2‐70.0
	Black	31.8	71.3	66.6‐75.5	35.1	54.5	48.8‐59.8
Cervix	White	~	~	~	20.4	49.2	44.2‐54.0
	Black	~	~	~	28.5	43.3	35.2‐51.1
Colon/Rectum	White	24.0	61.8	60.8‐62.8	21.2	60.4	59.4‐61.3
	Black	29.6	53.9	50.8‐56.9	34.7	57.6	55.4‐59.8
Corpus/Uterus	White	~	~	~	24.2	72.0	70.7‐73.3
	Black	~	~	~	39.4	52.6	48.9‐56.1
Female Breast ER‐	White	~	~	~	18.7	71.0	69‐72.9
	Black	~	~	~	35.5	64.8	60.9‐68.5
Female Breast ER+	White	~	~	~	18.7	87.9	87.3‐88.5
	Black	~	~	~	36.5	80.9	78.9‐82.8
Hodgkin Lymphoma	White	25.0	54.6	46.8‐61.8	23.9	54.3	46.3‐61.6
	Black	28.3	72.4	34.6‐90.6	38.5	57.1	31.8‐76.0
Liver	White	44.5	10.0	8.6‐11.6	39.4	9.2	7.3‐11.3
	Black	33.9	5.5	2.1‐11.4	41.3	10.5	4.9‐18.5
Myeloma	White	23.1	39.7	36.5‐42.9	19.5	32.3	28.9‐35.8
	Black	30.4	46.7	39.4‐53.7	31.8	42.1	36.0‐48.0
Non‐Hodgkin Lymphoma	White	23.4	54.8	53.1‐56.4	19.7	55.4	53.7‐57.0
	Black	28.5	52.1	44.4‐59.2	34.4	51.1	45.5‐56.5
Ovary	White	~	~	~	17.8	20.4	18.6‐22.3
	Black	~	~	~	35.4	19.2	14.9‐23.8
Pancreas	White	36.9	3.9	3.3‐4.6	31.5	4.1	3.5‐4.7
	Black	37.3	4.0	2.1‐6.8	48.9	1.9	1.1‐3.2
Prostate	White	17.9	91.6	91.2‐91.9	~	~	~
	Black	25.7	91.0	90.1‐91.7	~	~	~
Stomach	White	26.9	24.4	22.4‐26.5	25.9	31.8	29.3‐34.3
	Black	27.3	22.7	17.7‐28.1	37.2	31.8	27.1‐36.6

CI, confidence interval; ER, estrogen receptor; SEER, Surveillance, Epidemiology, and End Results.

Among both white and black males, the presence of diabetes was associated increased the risk of cancer death after controlling for age, stage, comorbidities, and treatments with an exception of Hodgkin lymphoma, liver, stomach, and bladder (in black males only) (Table 3). Blacks without or with diabetes have a higher risk of cancer death compared to whites with diabetes for most cancer sites, particularly bladder (black HR = 1.30, 1.30 compared to white HR = 1.17, *P* < .0001), colon/rectum (black HR = 1.24, 1.27, white HR = 1.16, *P* < .0001), liver (black HR = 1.23, 1.17, white HR = 1.03; *P* = .0004), NHL (black HR = 1.29, 1.53, white HR = 1.24, *P* < .0001), pancreas (black HR = 1.13, 1.17, white HR = 1.05, *P* < .0001), and stomach cancer (black HR = 1.12, 1.09, white HR = 1.04, *P* = .0076). As noted above, while males with diabetes were at slightly increased risk for mortality with most HRs less than 1.2. The highest relative increases in the risk of cancer‐specific death were observed in black males with diabetes and cancers of the bladder, colorectal, NHL, and prostate; ranging from 1.27 to 1.53.

**Table 3:** Hazard ratios of cancer‐specific deaths in males by cancer site, race, and diabetes status in SEER‐Medicare, 66+ y diagnosed in 2000‐2011

Cancer site	Race	Diabetes status	N	HR	95% CI	*P*‐values
Bladder	White	Without diabetes	32 207	Ref	~	<.0001
		With diabetes	10 426	1.17	1.14‐1.20	
	Black	Without diabetes	1107	1.30	1.21‐1.39	
		With diabetes	515	1.30	1.17‐1.44	
Colon/Rectum	White	Without diabetes	39 552	Ref	~	<.0001
		With diabetes	12 462	1.16	1.13‐1.19	
	Black	Without diabetes	3485	1.24	1.19‐1.29	
		With diabetes	1464	1.27	1.20‐1.35	
Hodgkin lymphoma	White	Without diabetes	683	Ref	~	.7236
		With diabetes	228	0.98	0.83‐1.17	
	Black	Without diabetes	38	0.89	0.60‐1.32	
		With diabetes	15	0.76	0.44‐1.30	
Liver	White	Without diabetes	3252	Ref	~	.0004
		With diabetes	2611	1.03	0.98‐1.09	
	Black	Without diabetes	411	1.23	1.11‐1.36	
		With diabetes	211	1.17	1.01‐1.34	
Myeloma	White	Without diabetes	5302	Ref	~	.0003
		With diabetes	1595	1.10	1.03‐1.17	
	Black	Without diabetes	784	1.11	1.02‐1.20	
		With diabetes	342	1.19	1.06‐1.34	
Non‐Hodgkin lymphoma	White	Without diabetes	15 336	Ref	~	<.0001
		With diabetes	4696	1.24	1.19‐1.29	
	Black	Without diabetes	621	1.29	1.18‐1.41	
		With diabetes	248	1.53	1.33‐1.76	
Pancreas	White	Without diabetes	8441	Ref	~	<.0001
		With diabetes	4944	1.05	1.02‐1.09	
	Black	Without diabetes	788	1.13	1.05‐1.22	
		With diabetes	468	1.17	1.07‐1.29	
Prostate	White	Without diabetes	141 215	Ref	~	<.0001
		With diabetes	30 745	1.22	1.20‐1.25	
	Black	Without diabetes	17 232	1.20	1.17‐1.22	
		With diabetes	5975	1.37	1.32‐1.42	
Stomach	White	Without diabetes	6465	Ref	~	.0076
		With diabetes	2384	1.04	0.99‐1.09	
	Black	Without diabetes	962	1.12	1.04‐1.20	
		With diabetes	361	1.09	0.98‐1.22	

CI, confidence interval; HR, hazard ratio; SEER, Surveillance, Epidemiology, and End Results.

All models adjusted for age, stage, comorbidities, and treatment.

The risks of cancer death were increased among females with diabetes across all cancer sites with the exception of stomach cancer (Table 4). Black women without or with diabetes have a higher risk of cancer death compared to white women with diabetes for some cancer sites, particularly bladder (black HR = 1.24, 1.32 compared to white HR = 1.21, *P* < .0001), corpus/uterus (black HR = 1.54, 1.66, white HR = 1.26, *P* < .0001), and ER negative female breast (black HR = 1.27, 1.29, white HR = 1.24, *P* < .0001). Cancer death risks for white women with diabetes were higher than those for black women only for myeloma. The highest relative death risks were observed among black women with diabetes for many cancer sites, with risks of 1.39 or greater for cancers of the corpus/uterus, ER positive breast cancer, Hodgkin's Lymphoma, NHL and ovary.

**Table 4:** Hazard ratios of cancer‐specific deaths in females by cancer site, race, and diabetes status in SEER‐Medicare, 66+ y diagnosed in 2000‐2011

Cancer site	Race	Diabetes status	N	HR	95% CI	*P*‐values
Bladder	White	Without diabetes	12 104	Ref	~	<.0001
		With diabetes	2844	1.21	1.16‐1.28	
	Black	Without diabetes	764	1.24	1.14‐1.36	
		With diabetes	414	1.32	1.18‐1.48	
Cervix	White	Without diabetes	2154	Ref	~	.0047
		With diabetes	553	1.19	1.07‐1.33	
	Black	Without diabetes	476	1.08	0.96‐1.21	
		With diabetes	190	1.18	1.00‐1.39	
Colon/Rectum	White	Without diabetes	48 648	Ref	~	<.0001
		With diabetes	13 112	1.19	1.16‐1.22	
	Black	Without diabetes	4693	1.14	1.10‐1.18	
		With diabetes	2493	1.21	1.16‐1.27	
Corpus/Uterus	White	Without diabetes	17 241	Ref	~	<.0001
		With diabetes	5502	1.26	1.22‐1.31	
	Black	Without diabetes	1518	1.54	1.44‐1.63	
		With diabetes	986	1.66	1.54‐1.79	
Female Breast ER ‐	White	Without diabetes	11 905	Ref	~	<.0001
		With diabetes	2737	1.24	1.18‐1.30	
	Black	Without diabetes	1402	1.27	1.19‐1.37	
		With diabetes	773	1.29	1.19‐1.41	
Female Breast ER +	White	Without diabetes	67 613	Ref	~	<.0001
		With diabetes	15 505	1.24	1.21‐1.27	
	Black	Without diabetes	3912	1.15	1.11‐1.21	
		With diabetes	2248	1.39	1.32‐1.47	
Hodgkin Lymphoma	White	Without diabetes	731	Ref	~	.0107
		With diabetes	229	1.27	1.07‐1.51	
	Black	Without diabetes	40	0.94	0.63‐1.41	
		With diabetes	25	1.62	1.02‐2.56	
Liver	White	Without diabetes	1989	Ref	~	.0099
		With diabetes	1293	1.09	1.02‐1.18	
	Black	Without diabetes	229	0.92	0.80‐1.06	
		With diabetes	161	1.18	1.00‐1.39	
Myeloma	White	Without diabetes	5168	Ref	~	<.0001
		With diabetes	1249	1.20	1.12‐1.28	
	Black	Without diabetes	989	1.01	0.94‐1.09	
		With diabetes	461	1.07	0.97‐1.19	
Non‐Hodgkin Lymphoma	White	Without diabetes	18 022	Ref	~	<.0001
		With diabetes	4415	1.29	1.24‐1.34	
	Black	Without diabetes	806	1.10	1.01‐1.20	
		With diabetes	422	1.44	1.29‐1.61	
Ovary	White	Without diabetes	11 738	Ref	~	<.0001
		With diabetes	2547	1.23	1.17‐1.29	
	Black	Without diabetes	733	1.22	1.13‐1.32	
		With diabetes	401	1.40	1.26‐1.56	
Pancreas	White	Without diabetes	11 699	Ref	~	<.0001
		With diabetes	5368	1.11	1.07‐1.15	
	Black	Without diabetes	1202	1.10	1.04‐1.17	
		With diabetes	1148	1.24	1.16‐1.31	
Stomach	White	Without diabetes	4806	Ref	~	.4339
		With diabetes	1681	0.97	0.91‐1.03	
	Black	Without diabetes	878	1.02	0.94‐1.10	
		With diabetes	519	1.05	0.95‐1.16	

CI, confidence interval; ER, estrogen receptor; HR, hazard ratio; SEER, Surveillance, Epidemiology, and End Results.

All models adjusted for age, stage, comorbidities, and treatment.

## 4 DISCUSSION

Overall, our study provides estimations of the extent to which coexisting diabetes increases risk of cancer‐specific death; it appears that this increased risk is present for most cancer sites. Furthermore, compared to white patients, black patients had higher death risks, particularly in the presence of diabetes for some cancer sites, particularly among women. The greatest increases in death risks were observed in black females with diabetes. However, the differing death risks by cancer site for white males and females compared to black males and females, suggest the need for further research to examine how prevalent diabetes can affect cancer deaths differently by race.

Several studies have investigated the association between preexisting diabetes and cancer mortality and were summarized in a recent meta‐analysis.^15^ This report included different types of studies involving small or selected patient populations, studies limited to one cancer site, or studies conducted outside the US. The study reported a 41% increased risk of mortality among cancer patients who had diabetes compared to those without diabetes across all cancer types.^15^ Their investigation also noted significant associations between the prevalence of diabetes and increased long‐term, all‐cause mortality and cancers of the endometrium, breast, and colon/rectum along with nonsignificant but elevated mortality risks for prostate, gastric, hepatocellular, lung, and pancreatic cancers. While limited to using Medicare patients older than 66 years, we extend this prior research by examining differences in the risks or cancer‐specific death by cancer and race for the top 14 cancer sites contributing to cancer mortality in the US through using a single large and more representative cohorts of cancer patients. Our cancer death risk estimates for specific cancers were similar to the meta‐analysis findings.

In general, our findings were consistent with other previous studies showing increased cancer death risks among black men across most cancer sites. These associations remained after controlling for the presence of diabetes, suggesting racial disparities between whites and blacks regardless of the presence of diabetes. The one exception was for Hodgkin lymphoma in which death risks were not different between white and black males, perhaps due to the limited number of cases. One prior study found higher rates of deaths in black patients with Hodgkin lymphoma primarily attributed to lower socioeconomic status among black cancer patients in the population‐based California Cancer Registry.^47^

With regard to cancer‐specific death risks among women, our findings supported previously reported associations between prevalent diabetes by race and cancer mortality risks with 2 notable exceptions. In our study, black females with and without prevalent diabetes had higher death risks compared to white women except for cervical cancer and myeloma in which white women with diabetes were at higher risk of mortality. One prior study has examined whether differences in mortality risk may be explained by the additional burden of comorbidities among black women with cervical cancer. It found that black women may be more likely to die from diabetes rather than cervical cancer; the authors suggested that the lower cervical cancer mortality observed among black women with cervical cancer was due to competing morbidities.^19^ We should also note that although black women had a higher prevalence of diabetes and the difference in prevalence between whites and blacks was greater than for male cancer patients, they did not experience markedly worse 5‐year survival rates as observed among colon/rectum, Hodgkin lymphoma, pancreas and stomach cancer patients.

Previous studies have suggested several explanations for the increased cancer‐specific deaths reported among cancer patients with diabetes. The association between diabetes and cancer may be direct as some evidence suggests that high levels of insulin or blood sugar in some diabetic patients may promote cancer cell growth due to increased tumor cell proliferation and metastases.^48^, ^49^, ^50^, ^51^ Hyperglycemia may increase endothelial cell permeability, effectively changing the structure of the cell and membrane making it more susceptible to metastases.^52^, ^53^, ^54^, ^55^ The relationship between diabetes and cancer may also be indirect, because diabetes and cancer share common risk factors such as age, sex, obesity, weight gain, poor diet, alcohol use, smoking, and physical inactivity,^45^ and some of these exposures may be more prevalent among blacks. Despite demographic and lifestyle risk factors having a higher impact on death risks for blacks, these risk factors may only partially explain the general pattern of increased cancer death risks for black populations,^19^, ^56^ and further research is needed to identify other unknown modifiable or biologic risk factors.

Interpretation of how diverse factors may underlie the association between diabetes and cancer deaths is further complicated by treatments used to manage these conditions. Prior research has examined whether differences in cancer treatment between patients with and without diabetes may explain the differences in mortality risks. Patients with diabetes may have other diabetes‐related comorbid conditions which may influence treatment plans,^51^ more limited response to cancer treatment, or be given less aggressive cancer treatments.^57^, ^58^, ^59^, ^60^, ^61^ It is possible that the diagnosis and treatment of cancer in diabetic patients may influence the management of their diabetes,^45^, ^51^, ^62^, ^63^ particularly with regard to medication intensity and adherence for black populations.^64^, ^65^ Existing racial disparities may be additionally worsened by difficulties in diagnosis, access to care, or access to follow‐up especially for blacks.^66^ While we were not able to investigate specific treatment‐related risk factors, there may be differences in how treatments are offered as well as differences in response to chemotherapy. It is possible that death risks may differ with regard to biological mechanisms and reactions to treatment by race. However, it was beyond the scope of our descriptive study to examine in detail the different effects by treatment. Future research including more detailed treatment data specific to each type of cancer is needed to explore that issue. Societal factors including education and socioeconomic status may also play a role in the observed health disparities regarding treatment, particularly among black women.^67^ Additionally, the increased risk of cancer deaths among diabetics has also been attributed to lower or suboptimal cancer screening rates.^57^, ^68^, ^69^ However, detailed data to examine those issues were not available in this study.

The main limitations in our study were the lack of biologic data, specific cancer‐related and diabetes‐related treatment, and lifestyle‐related health behaviors that may influence diabetes and cancer outcomes. We were able to adjust for stage and initial cancer treatment according to SEER data, although some components of cancer treatment, such as chemotherapy, hormonal therapy and some forms of radiation therapy, are underreported in these data.^70^ Therefore, we were not able to completely control for the effect of treatment. We were also not able to assess deaths due to competing risks, particularly death due to diabetes; therefore, our estimates may not accurately reflect the risks due to cancer‐specific deaths. However, this study provides a unique contribution in terms of the high‐quality surveillance data including deaths from SEER linked to the Medicare data to provide population‐based risk estimates of cancer‐specific deaths among diabetics and nondiabetics by race. Because the Medicare data are limited to adults 65 and older, our study may not be generalizable to younger cancer populations. However, this study provides for the first time, risk estimates on the independent and joint effects of diabetes and race on cancer deaths for several cohorts of cancer patients that is more representative of this age group of cancer patients in the US than has been provided in prior studies. We also show that while the differences in diabetes rates contribute to the differences in cancer death risks between whites and blacks, those differences do not fully explain why whites have different survival than blacks.

This study is the first descriptive study to investigate how the prevalence of diabetes affects population‐based cancer‐specific death risks for US cancer patients across several cancer sites by race and sex. Our study provides insight for new directions for future research to address racial disparities and to better understand how prevalent diabetes may affect cancer deaths differentially by race and sex. Diabetes prevalence is increasing, and these findings can provide important information to facilitate prevention efforts to reduce the burden of diabetes among cancer populations.

## CONFLICT OF INTEREST

There are no conflict of interest disclosures from any authors.
